# Hierarchical Name-Based Mechanism for Push-Data Broadcast Control in Information-Centric Multihop Wireless Networks

**DOI:** 10.3390/s19143034

**Published:** 2019-07-10

**Authors:** Rehmat Ullah, Muhammad Atif Ur Rehman, Byung Seo Kim

**Affiliations:** 1 Department of Electronics & Computer Engineering, Hongik University, Sejong City 30016, Korea; 2 Department of Software and Communications Engineering, Hongik University, Sejong City 30016, Korea

**Keywords:** named data networking, wireless sensor networks, sensors, push support, Internet of Things, message storming, Vehicular NDNs

## Abstract

By design, Named Data Networking (NDN) supports pull-based traffic, where content is retrieved only upon consumer request. However, some of the use cases (i.e., emergency situations) in the Internet of Things (IoT) requires push-based traffic, where a producer broadcasts the data based on the emergency situation without any consumer request. Therefore, it is necessary to modify the existing NDN forwarding engine when designing for an IoT scenario. Although solutions are provided to enable push-based traffic in IoT, the main solutions in the current literature lack data broadcast control design. Moreover, the existing solutions use an additional interest messages exchange, which creates extra overheads in the network, thereby resulting in higher delay and lower throughput. In this paper, therefore, we propose a name-based push-data broadcast control scheme for IoT systems, and consider two scenarios, i.e., smart buildings and vehicular networks. The proposed scheme consists of a robust content namespace design, device namespace design, and minor amendments to the data packet format and unsolicited data policy of the forwarding engine as well. The evaluation is carried out for both scenarios. Simulation experiments show that the proposed scheme outperforms the recent proposed schemes in terms of total number of data packets processed in the network, total energy consumption, and average delay in the network by varying the number of data packets per 2 s and varying vehicle speed.

## 1. Introduction

The Internet of Things (IoT) offers a vision in which the Internet expands into the real world, adopting everyday objects. The vision is based on an idea that the continuous developments in  information technology we have noticed in recent years will continue into the foreseen future. With the help of sensors, smart objects/things can understand the context, and through networking functions they can be able to connect with each other, gain access to Internet services and communicate with people. Due to the extensive access of sensors, it is estimated that the number of mobile nodes around the world will surpass 10 billion by 2020 and the global mobile data traffic will grow by more than 200 times from 2010 to 2020 [1]. To meet the requirements of IoT, advanced technologies are anticipated to alleviate the traffic load, reduce the delay, and support mobility and security. IoT is an enabler for services such as building automation, healthcare, smart cities, and smart grid, etc. However, the conventional IoT is built upon Internet Protocol (IP) architecture such as Advanced Message Queuing Protocol (AMQP) or Message Queue Telemetry Transport (MQTT) protocol which follows a client server approach, where a broker intervenes between producers and consumers [2]. In the conventional IoT design, the communication happens between fixed entities due to host-based approach i.e., TCP/IP (Transmission Control Protocol/Internet Protocol). Therefore, due to the rise of IoT it becomes challenging in terms of mobility, scalability, security, network management, and configuration. To mitigate the complexity of IP in IoT environment, it is of upmost importance to explore new computing paradigms to network communications.

Information-Centric Networking (ICN) is a promising paradigm that is based on named-based communication and the focus is on “What” not the “Where”. Named Data Networking (NDN) [3] (one of ICN architecture) is gaining more attention in the ICN community than other ICN architectures due to its simple communication model. NDN features such as naming data/objects, security, caching, and mobility support can bring manifold benefits to IoT applications. In NDN-IoT environment the content as well as the smart devices can be named and content can be fetched without knowing the location of the content providers. Most of the Internet traffic is content-based and the users are only interested in the content not the location of the content. NDN is based on two packet types, interest and data. The consumer(s) send interest packet(s) containing the names of the requested content and the data packet(s) flow back, carrying the named and secured content or chunks of the content, by following the same path through which the interest packets were sent. Three types of data structures are used in NDN on each node : (1) a content Store (CS), which caches the data subject to the caching policy; (2) a Pending Interest Table (PIT), which keeps records of the pending interest packets (3) a Forwarding Information Base (FIB), which directs interest packets towards the data providers. The detailed stepwise procedure is explained in Section 2. By design the communication process in NDN is pull-based where data is retrieved upon consumer request only. However, there may be cases where push-based data retrieval is required. That means the consumer does not need to request for the data, and the data will be sent to the consumer without consumer request. For instance, in the IoT cases, sometime the data must be sent to consumers without requests from consumers such as emergency alerts in case of some hazards. In the conventional NDN, any content router that receives content first checks its PIT. If there is no PIT entry for that content, the content is considered to be unsolicited (content which is not requested) and is dropped. Since the conventional NDN is natively pull-based model, therefore some amendments are required to enable push-based data delivery in NDN-based IoT. Therefore, in this paper, we enable name-based push-data broadcast control in NDN-IoT specifically for smart building case and we mitigate the data broadcast storm. Furthermore, we also design name-based push-data broadcast control for vehicular named data networks (VNDNs) to reduce the data broadcast storm.

As a summary, the major contributions of this paper are as follows:

1. A robust content namespace design and device namespace design for smart building to control the data packets flooding , thereby reducing the congestion in the network.
2. A robust content namespace design and vehicle namespace design for Vehicular NDN to control the data packets flooding.
3. Enable efficient data packet transfer to the appropriate consumer thereby reducing load on the network, minimizing energy consumption, and increasing throughput in the network.
4. Enhanced the conventional data packet format for marking the alert types in case of various emergency situations.
5. For evaluation of our scheme we consider two use cases (a) smart building case with static Mobile wireless nodes (b) VNDNs
6. Simulations in ndnSIM to check the performance of the proposed scheme with relevant schemes in terms of total number of data packets processed/forwarded in the network, total energy consumption by all nodes in the network and average delay.

The rest of the paper is organized as follows: The NDN background details are provided in  Section 2. Section 3 describes the challenges of IoT and the importance of NDN for IoT. The related work with limitations is explained in Section 4. The proposed method for smart building case and VNDNs is presented in Section 5. Section 6 provides the performance evaluations. Finally, Section 7  concludes this paper.

## 2. Background and Overview

### 2.1. NDN in a Nutshell

NDN is one of the famous and well-known architecture of ICN paradigm. Under ICN  paradigm, various types of architectures have been presented such as MobilityFirst, Network of  Information (NetInf), Data Oriented Network Architecture (DONA), Publish Subscribe Internet Technology (PURSUIT), content mediator architecture for content-aware networks (COMET) and CONVERGENCE [4]. The main idea is same in all architectures which is name-based network layer communication instead of IP addresses. ICN treats the data as a first-class citizen rather than the location of the data. Hence it is totally location independent and names are used for the data retrieval at the network layer. Before NDN, the *Content-Centric Network (CCN)* architecture [5] was coined by Van Jacobson as one of the projects at Palo Alto Research Center (PARC). CCN follows pull-based communication model in which the consumer request for data retrieval in the network. An interest is sent by a consumer which carries the name for the required content. The request is forwarded via all the intermediate content routers towards provider node. The provider then replies with a signed Content following the reverse path from which the content request was received. Any node on the path can reply with a Content if they have stored the content earlier. Furthermore, all the nodes on the path caches the content for future use so that the request for such content may not be forwarded to the original provider.

The CCN was then extended to NDN architecture and NDN also follows the interest and data exchange process for content retrieval. However, NDN is slightly different from CCN in architectural prospective. In CCN when a content request is received on a content router, then the very first operation is to check the CS. However, NDN checks the PIT at first and then the CS is checked. It is stated in the literature that the CS is much larger than PIT, therefore, to minimize the lookup delay NDN first checks the PIT [6].

### 2.2. The NDN forwarding Plane

**Figure 1:**
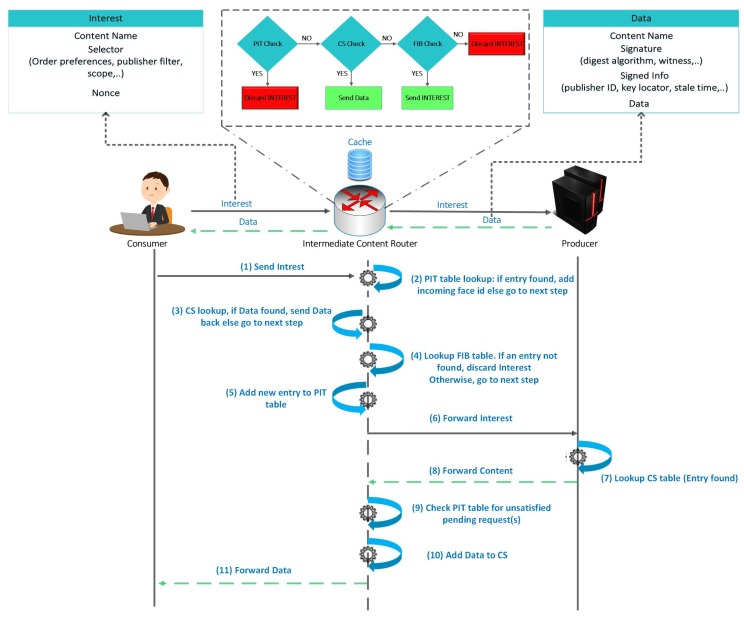
NDN communication process.

Figure 1 illustrates the stepwise communication process of an NDN architecture. To retrieve the content, a consumer sends an interest packet with the content name and its selectors (attributes, filters, resolutions, nonce etc.) When an interest packet is received by some intermediate content router, then the content router first checks the PIT. If an entry is found in PIT but with a different receiving face or with a different interest payload ID or with a different nonce, then the PIT table is updated by adding incoming interface entry and the packet is dropped. If an entry is not found in PIT, then the CS is checked using the content name and selectors. If the content is found, then the content is sent back to the interested consumer via an interface from which the request for content was received. If the content is not found in the CS, then a new PIT entry is created, and the interest packet is sent upstream via interface(s) stored in the FIB. The interest message is discarded if no FIB entry is found [7].

When a provider node or any intermediate node which has the cached content receives an interest packet, forwards the content with additional information to validate and verify the actual Content. The extra information added with the content include the cryptographic signature and the information about the signer etc. [6,8,9]. When the data packet is sent back to the consumer then the receiving node first checks the PIT. Upon successful entry match, the content is forwarded via stored interface(s) in PIT and stores the content in the CS subject to the caching policy. When the data is successfully forwarded then the corresponding PIT entry is deleted. The PIT entry is also deleted in a case if the data is not received within the lifetime of PIT entry. If no PIT entry is found for data, then the data is  dropped and considered to be unsolicited (data that is not requested before). Detailed processing steps of an interest packet and a data packet are depicted in Figure 1. In the following Section, we discuss the challenges of IoT network architecture and how ICN can be beneficial for IoT.

## 3. Challenges of IoT Network Architecture and Importance of ICN for IoT

The conventional Internet is based on TCP/IP model where the communication is carried between end-to-end hosts. However, due to massive increase in devices and data in IoT, the host-oriented model creates challenges in terms of scalability, mobility, security, and network management. The existing IoT architecture was not designed to fulfil the IoT requirements in an efficient way. To overcome the challenges of IoT due to TCP/IP, many solutions have been provided in research community. Among those solutions, NDN is one of the promising solutions for IoT and is considered to be one of the ideal candidates as argued in [10,11]. Some of the main challenges of conventional IoT the importance of NDN in the context of IoT are discussed as follows:

### 3.1. Scalability

IoT envisions that billions of devices will be connected to the Internet which creates challenges for the underlaying architecture in terms of scalability. The existing architecture produces Internet Protocol version 6 (IPv6) address spaces. Although IPV6 address space is a huge number, it is still a finite number which may create scalability and management issues in the future IoT. Furthermore, all these devices will generate a huge amount of data which is also a challenge in terms of network management and efficient scalability. To alleviate the scalability issues, ICN produces a novel feature of naming mechanisms. In NDN the data as well as the device can be named and can be accessed by expressing name of the content or name of the devices in interest message(s). There is no need to assign IP or numbering to devices. Moreover, as the current Internet users are more interested in data rather than location of the data, therefore, ICN naming mechanism(s) can be used to access the named data independently of its server location. Naming makes the data dissemination very efficient and result in lower content retrieval delay. The context (location, identity) and content is uniquely identified with names independently of the content location i.e., the IP address of the provider that host the content. Therefore, there is no need for end-to-end address mapping for content, context,  or services [12,13].

### 3.2. Caching

ICN provides in-network caching feature which reduces loads on the providers and brings content closer to the end users. This feature reduces the end-to-end mapping with servers as in the exiting IP. To be very specific, in IP networks if a user moves from one location to another location then it must establish a new connection with the server/provider. However, in NDN the content is cached at different locations along the path and can be fetched from the nearest cache node which alleviate the connection-oriented approach of IP networking. Moreover, the extended version of NDN named Named Function Networking (NFN) [14] provides named-based services fetching which means that the data and even results of functions/services can be cached along the path and will be available to other consumers without performing the computation again and again. Therefore, NDN not only provides content caching but also functions/code caching to avoid re-request the content or re-execute the function/code.

### 3.3. Receiver Driven Communication

NDN is basically a receiver driven communication model which means that communication initiates only when a consumer needs some data. As devices in IoT are resource constrained therefore, this feature is very beneficial for IoT scenarios to save the network resources since it can reduce the number of transmissions in the network. This is because the content will be transferred upon request only [15].

### 3.4. Mobility

In IoT a huge number of mobile devices will be connected to the Internet which may require anywhere and anytime connectivity. To make reliable connectivity and data availability anywhere, network architecture should support seamless mobility. As the conventional architecture i.e., TCP/IP is connection oriented and creates extra delays in the network when a mobile user moves in a network. In case of highly mobile networks such as vehicular ad hoc networks (VANETs), the connection between mobile nodes may be lost due to intermittent links. Therefore, if a node only receives the content from original server then every time the user must make a request in case of disruption. Whereas, in NDN, the consumer mobility support is natively provided . In case a mobile user moves to a new location then it will resend an interest packet for data. However, producer mobility is still under active research in NDN. Furthermore, NDN provides the caching of content on all nodes, thus there is no need to request the original server for the content repeatedly. Instead it can be retrieved from the nearest nodes that has the cached content [16].

### 3.5. Security

The conventional Internet architecture secures the channel over the network and many solutions are provided to cater the security issues such as Internet Protocol Security (IPsec). However, the security approaches used in existing Internet architecture is not good fit for IoT [17]. Securing a channel or session between devices does not tackle the heterogeneity issue of IoT. As smart sensors are the building blocks of IoT which are heterogeneous in nature and vary in terms of memory, size, processing power, and battery life. Furthermore, the communication between such devices is carried out via various underlying technologies (i.e., wired, Bluetooth, Wi-Fi, cellular, fourth generation (4G), Long-Term Evolution (LTE) etc.). These all different interfaces require different networking stacks and each stack has different security solutions. However, in NDN protection and trust are implemented at the  packet level, rather than at the channel level. By design, NDN offers native support for security, which are still not effectively available in the host-centric paradigm. In NDN each packet is  signed at the time of production, cryptographically binding the names and the payload of the data. Information about the signing key i.e., the name of the signing key certificate is recorded in key locater field of the data packet. Therefore, NDN allows applications in IoT to freely distribute data in the network without requiring any kind of trust at intermediate nodes to keep data secure.

### 3.6. Reliability

Presently, most of the Internet users are interested in the content not the location of the content/data. The conventional Internet is based on TCP/IP model which cares about the location of the data and the main purpose of this design was to share the expensive resources of the Internet. However, the today’s Internet is not limited to share network resources but to disseminate information in a fast manner to billions of devices/users. In this context, the NDN paradigm has been proposed which promises to resolve such issues via naming the data. The reliable data transfer used in IP Internet is not good fit for IoT applications because of various constraints of IoT devices such as battery, and memory etc. and the traffic generated by these devices. Therefore, battery constrained devices use on-off cycles to extend their battery lives. The frequent connection establishment induced extra latencies in TCP/IP. However, the naming in NDN makes the content easy to search and retrieve. The in-networking feature of NDN decreases the frequency of fetching data from the provider and thus prolongs the network lifetime. Moreover, the physical layer effects of wireless links can make the functioning of TCP worse. However, NDN split the transport layer functionalities between application layer and NDN forwarding layer. Therefore, the routers are responsible for maintaining per interest forwarding state for error recovery and retransmission [15,18].

## 4. Related Work and Limitations

NDN has been actively researched in the areas such as IoT, wireless sensor networks (WSN), VANETs, and smart grid, etc. WSN is an integral part of IoT where large number of battery power devices are deployed for sensing and communication. Mostly such devices are installed in some specific sensing areas for monitoring purposes, i.e.,  temperature, pressure, humidity, etc. One of the main objectives of WSN is monitoring an area which needs long life battery timings. The battery is consumed in WSN via, sensing, processing, and listening. Therefore, each node in WSN should effectively operate to prolong its battery lifetime. In this regard, authors in [19] employed NDN in WSNs using hierarchical naming scheme, where the interest and data messages of 102 bytes are used to match with the IEEE 802.15.4 frame and all the processing steps of these messages are modified to suit the processing capabilities of the WNS nodes. The authors in [20] implemented the CCN-communication architecture for WSN and is termed as “CCN-WSN”. The implementation is done via hierarchical naming, and the data structure of NDN i.e., PIT, CS, and FIB are implemented. In [21] the NDN application is exploited in WSN. The authors produce a defer-window concept. The collision issue due to NDN broadcast is alleviated via defer window during the interest and data packet transmission.

The authors in [22] proposed a scheme for NDN in IoT. The authors claim that it is difficult to implement the complete NDN mechanism for IoT nodes due to its resource constrained nature in terms of battery, memory, storage etc. Therefore, some feature such as security and caching are handled by the third-party nodes. They have considered the IoT environment and evaluate it in terms of energy consumption and bandwidth. For benchmark work they compared their scheme with IP-based communication.

In [23] authors implemented NDN in IoT for building scenario. For the implementation they have used CCN-Lite [24] over the RIOT [25]. In the evaluation, a “Vanilla Interest Flooding” (VIF) has been used where the nodes flood the interest based on interest flooding scheme. One producer and many consumers has been considered in the network and the assumption is made that the content is only hosted at single provider which may not be a general case. The analysis is carried out with caching and no caching using single and multiple consumers. For multiple data sources authors in [26] uses NDN for an IoT scenario. A new interest packet is introduced that requests data from multiple sources, named multisource Interest (msINT). For collision-avoidance they have used the concept of contention window. The data source defers its transmission until the collision-avoidance random period and the CWmax is dynamically adjusted based on the number of the data sources. Recently NDN naming schemes has been adopted for many IoT scenarios such as smart home [27], VANETs [28], smart building [29], underwater networks [30] and smart campus [31].

Most of the NDN work in the context of IoT have focused a scenario where an interest is used to subscribe for data. The consumers then receive the data via subscription. This is good for the IoT applications which are periodic; however, the data may also be generated in case of  emergency or an event detection. By design NDN is pull-based and the emergency data is  considered to be unsolicited data (data which is not requested) and such unsolicited data is discarded in NDN. Therefore, the application in IoT also requires this data to be pushed into the network.

For handling push-based data transfer in NDN, related work has been proposed with various approaches. In [32], ICN architecture for IoT has been proposed. They considered the home automation system scenario as a use case and they have used the push-based scenario in their implementation for home automation system. In [33], the authors proposed a push-based communication system for IoT where they emphasis on the subscription scenario in IoT. For subscription, they sent an interest message with NDN hierarchal naming scheme without creating PIT entries. The reason not to create PIT entry is that there may not be the immediate data available for the interests. The data will be sent to the subscribers whenever it is available. As the PIT entries avoid looping, therefore, authors in [33] uses a timestamp in each content name to avoid the routing loops. The authors in [34] proposed a  scheme of pre-caching in which the unsolicited data is handled via temporary interest packets so that it is not discarded on arrival by the intermediate content routers. This scheme brings additional overhead with two-way handshake and using an additional payload field. In [35], authors proposed Long-lived interest scheme for unsolicited data retrieval. However, Long-lived interest packets lock PIT entries for longer periods of time. The authors in [36] proposed a lighter version of beacon-based push support known as beacon alert message (BAM) which was created using fixed sequence number. However, authors used the two-way handshake for creating synthetic PIT entries. In [37], authors  proposed a beacon messages-based push support in VNDNs. However, the beacon messages are forwarded in a broadcast manner towards the destined consumer. As the nature of wireless is broadcast, therefore, multiple nodes may receive the beacons in the radius of provider node, thus result in data broadcast storm. The data packet is larger than interest packet due to carrying the actual content. Therefore, it causes more congestion and waste the bandwidth and other resources of the network. Therefore, this flooding result in bandwidth wastage, battery issue of the wireless network, delay, redundancy, and so on.

Most of the existing works enabled the push-based data transfer in NDN using beacon messages, interest notifications, and long-lived interest. However, none of the existing Push-based schemes are proposed that control the data broadcast storm via naming in the push-based scenarios. Therefore, in order to reduce the data broadcast storm, we propose a named-based push-data broadcast control scheme that limits the push-based data flooding towards consumer in IoT networks. Moreover, we are not using any beacon messages, long-lived interest, and interest notification for enabling push-based traffic in NDN-IoT. Furthermore, as the NDN is also applied to VANETs in recent, therefore, we also design name-based push-data broadcast control scheme for VNDNs. In the following Section, first we explain our proposed naming scheme for smart building scenario. After that, the proposed naming scheme for VNDNs is presented.

## 5. Proposed Scheme

In this section, to clarify the features of the proposed scheme, a motivation of this paper is provided. After that, a proposed scheme is presented for smart building.

### 5.1. Motivation

**Figure 2:**
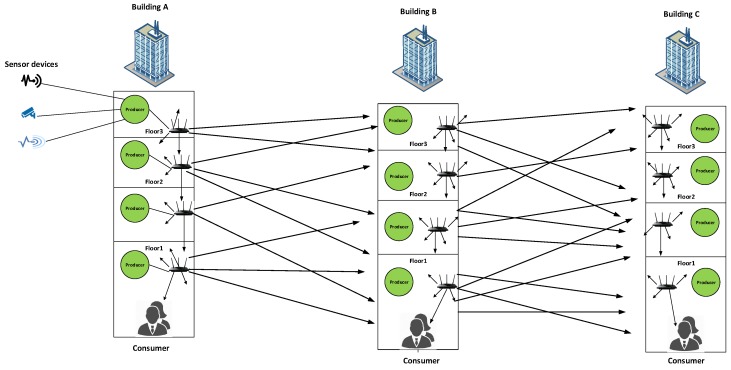
Problem Scenario.

The motivation might be illustrated by using an example shown in Figure 2 which shows a use-case scenario of a smart campus. Figure 2 illustrates that there are three buildings i.e., A, B, and C. Each building comprises of three floors and each floor has multiple rooms. In our use case we assume that each room on every floor has various type of sensor nodes such as temperature, camera, fire, and smoke sensors. All the sensor nodes in each room is connected with one producer node of that room which collects data from all the sensor nodes and then forward it towards the consumers. Each floor comprises of Producer nodes and a Wi-Fi access points. Each building has a Consumer node which controls/monitors the relevant building. That means if something unusual occurs in the building such as fire, or any intruder activity then the consumer node will be informed on emergency basis with lower delay.

Let’s assume that an emergency situation occurs at the third floor of building A. Now the producer node will not wait for a consumer request and it will broadcast the data in the transmission range instead. When the data is broadcasted then all the devices (Wi-Fi) in the transmission range will also broadcast the data. Figure 2 illustrates that a producer at the third floor of building A broadcasts an emergency alert. This emergency alert has been received by all devices in the transmission range including building B. When the building B devices receive this data, then it will be rebroadcasted in the transmission range and eventually it will be flooded in all buildings of the campus. Although the data is only destined for the consumer in building A, it is flooded in all the buildings of the campus. This flooding creates congestion and overhead in the network which directly affects the delay, throughput, and other resources of the network. As a result, the emergency data might not reach on proper time to the appropriate consumer to take timely action. This data flooding is a serious problem in IoT network where forwarding of time critical information is the first priority. So far, there are no such mechanisms available in the literature (to the best of our knowledge) that address these issues via NDN namespace design. Therefore, we propose a namespace design for content and device as well to mitigate and control the data flooding.

### 5.2. Proposed Scheme Description

To control the push-based data in IoT, we proposed a naming scheme comprises of data namespace design, device namespace design, and enhancement to the conventional data packet for tackling the type of alerts. Before we discuss the content and device namespace design, it is important to elaborate on the amendment of conventional data packet and the changes of class for unsolicited data that abstract NDN data packets.

#### 5.2.1. Enhanced Data Packet Format

**Figure 3:**
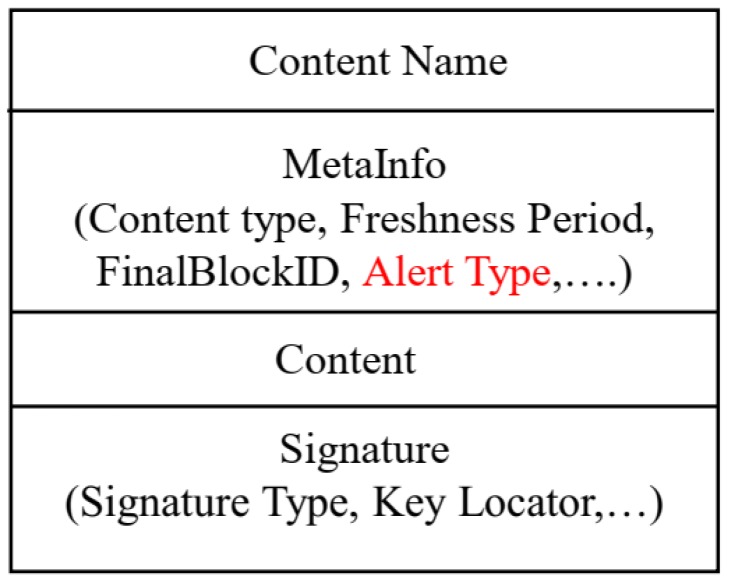
Enhanced Data Packet Format.

We performed a minor modification of the NDN packet format in the optional information field (MetaInfo) with “AlertType ” for marking the types of alert. Since there could be several types of alert in a smart campus scenario such as smoke alert, heat alert, fire alert (gives early warning of developing fires to enable evacuation), and intruder alert (detects attempted intrusion or unauthorized entry into a building or room and such alarms may be linked to surveillance cameras, light systems, and remote monitoring by security companies). For instance, when temperature increases than a certain threshold in a room, then the concerned person or administrator of that building or floor must be alerted. Data packets generated by that room gets named by the name of the room where the hazard occurs. The enhanced data packet format is shown in Figure 3. The changes of class for unsolicited data that abstract NDN data packets are detailed in Section 6.

#### 5.2.2. Data/Content Namespace Design

In our naming scheme the data packets are limited to a specific building in a smart campus and the design follows hierarchical naming structure of NDN. Our  generic namespace design comprises of various components in a hierarchical structure, i.e., /Campus/BuildingName/FloorNumber/ RoomNumber/SensorId payload, where campus shows the campus name or location, building shows the building name, floor number is the specific floor number in a building, room number is the specific room number in a building , sensor Id shows the id of sensor and payload carries the actual content. More specifically, we are considering a smart campus scenario of our university (Hongik University). For instance, a namespace of a building A in Hongik University would be /Hongik/BuildingA/Floor3/Room301/Id001 Alert. This namespace shows that an alert has been triggered in the Hongik University and in the third floor of building A in room number 301. This alert which actually represents data must reach the consumer node (which would be an administrator that controls the building) of the building A, to inform the concerned consumer for taking action accordingly. The consumer receives the data packet with its name and all the other optional fields. Since we are using metainfo field of the data packet by marking the alert type. Therefore, the consumer could easily get the information about the type of alert received. It is to be noted that the conventional NDN is natively pull-based model, therefore some amendments are required to enable push-based data delivery in NDN-based IoT and are detailed in Section 6. Moreover, we assume that all the devices are authorized. In push-based NDN communications all the data packets are broadcasted. For instance, when building A broadcasts data packets then all the nearby devices in the range of building A (i.e., building B or C) also receive the data packets. Therefore, all the nearby buildings in the range will broadcast the data packets which creates extra overhead in the network and the data packets might not reach to the appropriate consumer on appropriate time. To control such data packets broadcast storm there is need for namespace design for device as well.

#### 5.2.3. Device Namespace Design

**Figure 4:**
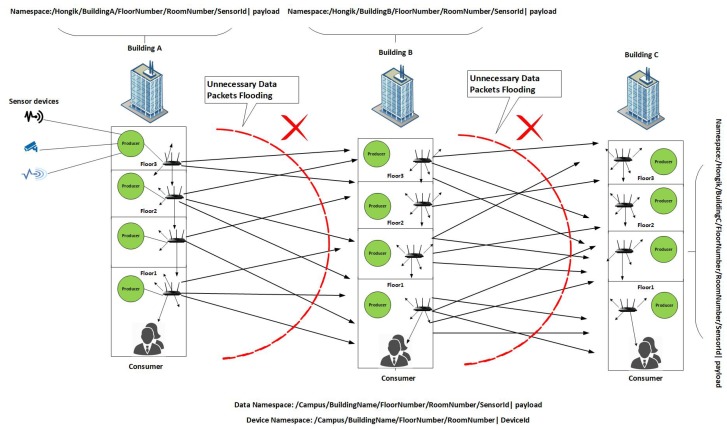
Proposed Scheme.

We also design the hierarchical namespace for devices in a smart campus. For example, the device of the third floor of building A is named as /Hongik/BuildingA/Floor3/Room301/Device005. The namespace shows that device 005 belongs to third floor of building A located at Hongik University in room number 301. Therefore, when the data packet is broadcasted from building A, then it will be received by all the devices in the transmission range of building A. Each device when receives the data packet checks the namespace of the received data packet and compare with its own namespace. If the first two components of the namespace of data packet, such as /Hongik/BuildingA/ matches with the first two components of the namespace of device such as /Hongik/BuildingA/, then the data packet is processed and is forwarded further. The data packet is relayed via all the intermediate devices of the building A from floor 3→ floor 2 → floor 1 and eventually it reaches to the consumer node where appropriate action might be taken accordingly. It is to be noted that the consumer is located at the first floor of each building as depicted in Figure 4. In case the namespace of data packet and the namespace of device does not match then the data packet is discarded and does not forwarded further. Algorithm 1 shows working of the proposed scheme. Let’s suppose that a device of building B receives the broadcasted data packets from building A then the devices of building B compare their namespaces with the received data packet namespaces. Since the first two components of namespace i.e., /Hongik/BuildingA/ shows that the data packet is destined for a consumer of building A and not for building B. Therefore, all the devices of building B will discard the data packets and will not process/forward the data/content further as shown in Figure 4. In this way the data packet flooding is controlled and not flooded further in the whole campus. In our case, we assume that the destined consumer is in the same building where the provider is located. In addition, if the  data is destined to some other consumer in other building, then source and destination information in the data packet will be checked accordingly.

Algorithm 1 shows the critical data delivery in NDN-based network. However, it does not mean that our scheme does not work for pull-based communication. The pull-based mechanism works in normal network scenario whereas the push-based operation is triggered whenever any emergency occurs, and the critical data needs to be delivered in a fast manner. For that we have enabled push-based data delivery by changing the Networking Forwarding Daemon (NFD) and we assume that all nodes in the network are authorized. The assumption is taken to check the effectiveness of our proposed naming scheme in emergency situations. That means if an emergency occurs then the producer does not need wait for consumer’s request and the emergency data needs to be delivered promptly. In a normal case, when the data is not requested then data is considered to be unsolicited data and dropped. Hence in our case the data will not be dropped and will be accepted but with the proposed algorithm logic. Such data when received on a node, then our algorithm first checks the data whether it is unsolicited or not. By unsolicited data we are meaning that there is no entry found in the PIT table. Therefore, when such data arrives on a node then the receiving node will check their PIT entry corresponding to that data. If no entry founds, then the data is considered to be unsolicited data. After that, our proposed naming scheme will be employed whether to accept the data or drop the data by comparing the device namespace with the data namespace. If the first two component of the data namespace matches with the first two components of the device namespace that means the data is destined for that building and need to be processed. The data will be saved in the CS and will be forwarded further if necessary. Moreover, if the data is not unsolicited that means there is an entry available in the PIT table corresponding to that data. Hence, the conventional NDN operation will be followed. In that case the PIT entries have been checked and entry has been found for that data. Therefore, it depends on the number entries (one or more) in PIT. Based on these entries, the data packet is then forwarded to the InFace(s) which is available in PIT. Moreover, the data is saved in the content store of the nodes bases on the caching policies and the name, nonce, and face(s) information is then deleted from the PIT.   

**Algorithm 1** Received data packet in the Proposed Push-based Scheme for Building Scenario**procedure** Received [Name, MetaInfo, Data, Signature] Check PIT for Unsolicited Data    **if** (Data is Unsolicited()) **then**        CompareDatanamespacewithDevicenamespace;        Data.Name←*First two components of Data;*        Device.Name←*First two components of Device;*        **if** (Data.Name equals Device.Name ) **then**           InserttheDataPacketinCS;           ForwardtheDataPacket;        **else**           DiscardtheDataPacket;        **end if**    **else**        **if** (If face is application) **then**           ReceivedtheDataPacket;        **else**           ForwardDataPackettoface;           Remove[Name,Nonce,Face]fromPIT;        **end if**        getPendingDownstreamFace;    **end if** **end procedure**

### 5.3. Proposed Scheme for Vehicular Scenario

Recently NDN has been introduced in VANETs to change the host-oriented model of VANETs from IP to named data communication [6]. In VNDNs, consumer follows pull-based mechanism where a consumer sends an interest packet for content retrieval. However, the pull-based communication model of VNDNs proposals to date introduce overhead in the network by using additional messages such as beacons , especially when considering critical data that need to be forwarded immediately. Recent work has proposed methods to address this issue in VNDNs. Author in [37] propose a mechanism for push-based data dissemination for VNDNs in which a producer node can inject content through beacon messages. The author proposal relies on beacon messages to assist the forwarding of data packets. Moreover, the beacon messages are broadcasted which is also creating congestion. Although the beacon messages are of small sizes, it still creates congestion in the network due to broadcast. As the beacons carry control information, therefore, broadcasting beacons in VNDNs may result in the broadcast storm and high bandwidth usage. As a result, the network is overloaded. The existing schemes in VNDNs control the interest broadcast storm via hop limits and with other extra controlling information which brings overhead to the network. For this reason, we introduce a name-based data broadcast control mechanism for VNDNs. Our scheme is different from others in a sense that we do not use extra control messages such as beacons. Our scheme is based on Global Positioning System (GPS) location and the vehicles get its location information via GPS for data packet transfer. In our scheme, the vehicle sends data packets to only the destined consumer (Road-Side Unit (RSU) ) in the region, which helps to reduce the number of data packets in the network. The detailed process of the proposed name-based broadcast control in explained in the following subsection. Before we explain our proposed scheme, we would like to highlight the limitations of the  proposed scheme in [37] which brings following challenges for VNDNs. After that, the proposed scheme is presented.

- In the realistic scenario, when an accident happens, then the producer node (accidental vehicle) start sending emergency messages to the nearby RSU. Therefore, the producer node in case of an accident must be immovable due to an accident. However, authors in [37] claimed that the producer node is mobile and sending emergency notifications to the nearest RSU, which is not a realistic case. In our scheme the producer node is immovable and sends emergency information to all the intermediate vehicles.
- As the vehicles in VANETs are mobile and move with a certain speed. Therefore, the intermittent connectivity happens between nodes. During mobility, the vehicles quickly move out of the range easily. The authors in [37] did not define how to cope with this issue. In their scheme, the beacons are sent for making synthetic PIT entries on nodes for subsequent data arrival; however, they did not define a certain time of how long the vehicle may stay in a specific region. That means if a beacon reaches to a vehicle in order to make PIT entry, the vehicle may move out of the range after some time. In that case, the subsequent data packet will be considered to be unsolicited and will be dropped. If there is not a certain time defined for beacons and vehicles, then the purpose of sending beacons for making PIT entries are useless and it works only for static cases. This issue is addressed by [38] where authors defined a certain link layer threshold. If that threshold meets then the packet will be processed. Otherwise vehicles will not forward packets, since the vehicle may not connect or may not in the range. In our scheme, we use the scheme defined in [38] to deal with intermittent connectivity due to fast mobility of vehicles.
- Moreover, in [37] the beacons are sent in a broadcast manner which increases the broadcast storm in the network. According to the NDN rituals the beacons will also be considered to be interest or data packets. In our case, our scheme requires neither any synthetic PIT entries nor any additional beacon messages.

Our proposed scheme mitigates the above-listed shortcomings and is detailed as follows:

#### 5.3.1. Proposed Scheme Description

**Figure 5:**
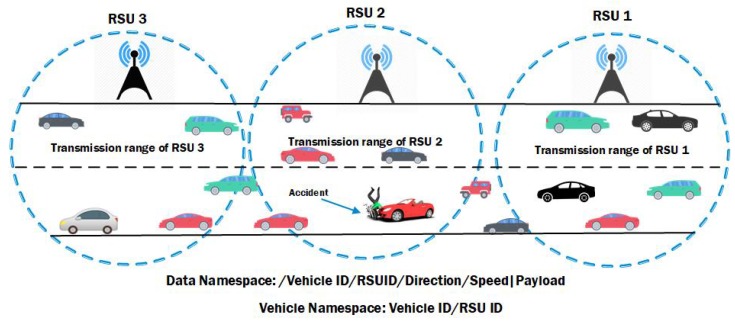
Vehicular Named Data Networks (VNDNs).

We have proposed a variable naming scheme for VNDNs that efficiently reduces flooding of  data packets, thus optimizing network use. To explain our naming scheme for vehicular scenario, we consider an emergency(accident) situation. Figure 5 presents a scenario in which the scheme is carried out. Figure 5 describes a situation where a vehicle broadcasts data packets in case of an accident to all nearby vehicles. All the nearby vehicles relay the data packet to the appropriate consumer which is an RSU in our case. We assumed that each vehicle is equipped with GPS and is aware of its own location information as used by modern automotive navigation systems. The vehicles positions are obtained locally through GPS. Each node’s position is sent in the data packet right after the prefix name. The other fields remain the same of NDN data. Hence every time a relaying vehicle forward a data packet, it first verifies its current position from its local GPS and afterwards includes such information.

The uniqueness of our naming scheme is variable names. As the VANETs are completely mobile, therefore to use a static/constant naming scheme for such high dynamic network may not work. We should have a variable naming scheme that change the data namespace based on the location and regions. Moreover, in our scheme the communication is Vehicle to Vehicle (V2V), and the vehicles are intermediate nodes which act as relay nodes to forward data to the consumers (RSUs). These intermediate nodes are the same as the case of the building scenario; however, in this case all the nodes are mobile.

#### 5.3.2. Data and Vehicle Namespace Design for VNDNs

The namespace design for VNDNs follows hierarchical naming structure of NDN consisting of  different attributes separated by “/”. Our generic namespace design comprises of various components in a hierarchical structure, i.e., /Vehicle ID/RSUID/Direction/Speed payload, where Vehicle ID shows the unique ID of a vehicle, RSU ID shows the ID of a vehicle which changes with GPS, direction shows the vehicle direction, speed shows the vehicle speed, and the payload carries the actual data.

Let’s suppose that an accident occurs near RSU2. That means the accidental vehicle is in the range of RSU2. In that case, the affected vehicle (producer) will not wait for a consumer request and it will broadcast the data immediately in the transmission range instead. Due to ad hoc scenario, the data will be broadcasted in the transmission range and all vehicles will broadcast the data packets further. Figure 5 illustrates that a producer node (accidental vehicle) which is in the transmission range of RSU2 broadcasts an emergency alert. This emergency alert has been received by all vehicles in the transmission range. Since these vehicles are moving therefore, they will move into other RSUs range and will further broadcast the data in their transmission range. In this way, all the vehicles start transmission of data packets which will be flooded in the network. Although the data packet is destined for RSU2, it is flooded to all RSUs. Such flooding creates congestion and overhead in the network which directly affects the resources of the network. As a result, the emergency data might not reach on proper time to the appropriate consumer to take timely action. This data flooding is a serious issue in VNDNs where forwarding of critical data is first priority. Most of the papers in the VNDNs literature mitigated this issue via interest suppression schemes and other metrics such as hop counts etc. So far, there are no such mechanisms available in the literature for VNDNs that address this issue via NDN namespace design.

In this paper, for the first time, we propose a namespace design for push-based critical data control in VNDNs. We believe that if a vehicle is mobile then the vehicle name should also be variable with mobility. Therefore, our namespace is variable, and the data name is variable based on the GPS location of the vehicle. For instance, an accident happened in the transmission range of RSU2 and the vehicle id is 5. We assume that vehicle is moving in the east direction and its position is in the RSU2’s range. The accidental vehicle generates a data packet and broadcasts in its range. Therefore, the namespace of a Vehicle would be /VehicleID5/RSU2 /East/X,Y/50Kmph|accident. When the data packet is broadcasted by Vehicle with ID 5, then it will be received by all the vehicles in the transmission range of vehicle 5. Each vehicle when receives the data packet first checks the namespace of the received data packet and compares with its own namespace. The vehicle namespace would be /Vehicle ID/RSUID. This namespace shows that the accidental vehicle id is 5 and this vehicle is moving in the east direction and in the range of RSU2. The namespace indicates that the data packet is destined for RSU2. If the second component of the data namespace i.e.,  /RSU2 matches with the second component of the Vehicle namespace i.e.,  /RSU2, then the data packet is processed and relayed via all the intermediate vehicles of the RSU2. In case the data packet namespace and the vehicle namespace does not match then the data packet is discarded and does not forwarded further. Moreover, if a vehicle receives the broadcasted data packets from an accidental vehicle in the range of RSU2 and the vehicle moves out of the transmission range of RSU 2 and enters RSU3. Now in normal case this vehicle and other vehicles also broadcast and carry the data packet which result in a broadcast storm of data. However, in our case, the other vehicles in the range of RSU3 when receive the data packet, first compare the namespaces with the received data packet namespaces. As the second component of namespace i-/Vehicle id5/RSU2 / shows that the data packet is destined for an RSU2 and not for RSU3. Therefore, all the vehicles of RSU3 discard the data packets and not process/forward the data packet further. In this way, the data packet flooding is controlled. Algorithm 2 shows working of the proposed scheme.    

**Algorithm 2** Received data packet in the Proposed Push-based Scheme for VNDNs**procedure** Received [Name, MetaInfo, Data, Signature] Check PIT for Unsolicited Data    **if** (Data is Unsolicited()) **then**        CompareDatanamespacewithVehiclenamespace;        Data.Name←*Second component of Data;*        Vehicle.Name←*Second component of Vehicle;*        **if** (Data.Name equals Vehicle.Name ) **then**           InserttheDataPacketinCS;           ForwardtheDataPacket;        **else**           DiscardtheDataPacket;        **end if**    **else**        **if** (If face is application) **then**           RecievedtheDataPacket;        **else**           ForwardDataPackettoface;           Remove[Name,Nonce,Face]fromPIT;        **end if**        getPendingDownstreamFace;    **end if** **end procedure**

## 6. Performance Evaluations

### 6.1. Performance Evaluation Metrics

We conduct several experiments to evaluate the performance of the proposed as well as the benchmark protocol using following metrics.

#### 6.1.1. Number of Data Packets Processed (DPP)

Overall number of data packets processed is defined as the total number of data packets transmitted in the network to the total number of generated data packets by the producer node and relay nodes and can be defined as follows:

(1) $$\mathrm{DPP}=\frac{\sum_{1}^{N}TransmittedDataPackets}{\sum_{1}^{n}GeneratedDataPackets}$$

where “*n*” is the total number of generated data packets and “*N*” is the total number of transmitted data packets in the network.

#### 6.1.2. Total Energy Consumption

Energy consumptions of a single node depends on two major factors (1) The energy required to process a data packet, and (2) reception of a data packet and transmission of a data packet. We adopt the energy model used in [39] to evaluate the power consumption in the simulation experiments. The overall energy consumption of a single node can be defined as follows:

(2) Enode=EProcessing+EReception+ETransmission

The overall energy consumption of a network is directly proportional to the number of packets transmitted in the network and can be defined as follows.

(3) Enetwork=∑1pEProcessing+EReception+ETransmission

where “*p*” is the total number of packets in the network. Moreover, the initial energy is 10 Joules and the energy consumption per bit is 0.5 μJ.

#### 6.1.3. Average Delay

The average delay is the time taken from provider node to the consumer node travelled by the data packet in a network. The time when the data packets are transmitted from the provider node is noted and when it arrives at the consumer node and then its arrival time is calculated and is defined as follows:

(4) $$\mathrm{Total}Delay=\sum_{n=1}^{n}\left(ReceivedTime-TransmitTime\right)$$

(5) $$\mathrm{Average}Delay=\frac{TotalDelay}{TotalPacketsReceived}$$

where “*n*” is the total number of generated data packets in the network.

### 6.2. Simulation Environment: Smart Building Scenario

**Figure 6:**
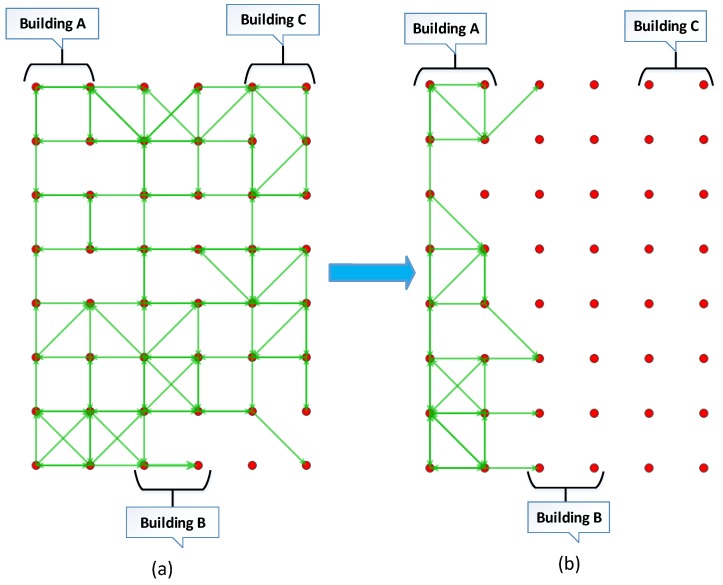
Simulation Scenario for smart building: (**a**) Simulation scenario with NDN-DISCA, (**b**) Simulation scenario with proposed scheme NDN-NDBC.

The performances of the proposed method “Hierarchical Name-based push-Data Broadcast Control” called NDN-NBDC, are comparatively evaluated with [36] called NDN-DISCA and [40] called NDN-BBCD. We considered it as a baseline for our evaluation. The result in this paper are based on a modified codebase of NDN in ndnSIM and NFD, which was implemented for pull-based data retrieval [41]. We used a grid topology for our simulations as shown in Figure 6a,b. Figure 6a depicts the NDN-DISCA scenario, where the beacon alerts are broadcasted and Figure 6b illustrates our proposed scenario. To enable unsolicited data in push-based NDN, we performed a minor change in NFD and ndnSIM simulator. We initialized a Boolean variable “*is-data-unsolicited*” to true and make changes on the class that abstract the data packets. Therefore, when the data packet is received on a node, then all the PIT entries are checked for the name of the received data packet. If there is no match found in the PIT entries for the data packet received, then the Boolean “*is-data-unsolicited*” value remains true which means the data packet is unsolicited. Therefore, the data packet will be cached and processed. After cache, the data will be forwarded on the node’s faces. It is to be noted that our scheme is based on an assumption that all the nodes are authorized. If the Boolean “*is-data-unsolicited*” value becomes false that means the data packet has been requested and an entry in the PIT has been found. Therefore, the conventional NDN operation will be followed [42]. It is evident form Figure 6a that if the data packets are not controlled in push-based scenarios then it creates a data broadcast storm in the network and each node within the transmission range start broadcasting the data packets. Whereas in our proposed scheme the data packets are limited to a specific building as depicted in Figure 6b. In the simulations, the data packet size is 1040 bytes and IEEE 802.11a standard [43] is adopted for physical and data link layers. The Constant-Speed Propagation Delay Model on the NS-3 simulator was used for the Propagation Delay Model. The LogDistance-PropagationLossModel of the NS-3 simulator was applied as the Propagation Loss Model. Each experiment was conducted 4 times using the seed value, and the results were acquired from the average values of the measurements. The main simulation parameters are summarized in Table 1.

Figure 6a,b shows the location of building A, building B and building C on a 40 × 40 grid topology that is made up of 48 static Nodes. Each building comprises of 16 static nodes including one provider node and one consumer node. We also deploy four mobile nodes in the building to exhibit the mobile behavior of nodes and for carrying the data packets to consumers.

### 6.3. Performance Analysis

Our first benchmark scheme is NDN-DISCA [36] that is a beacon-based push support which creates BAM using fixed sequence number. In this mechanism, the BAMs are sent in a broadcast manner towards the destined consumer. It enables the push-based data delivery without the request from consumer. However, for creating entries in the PIT the beacon alert messages are used. As the nature of wireless is broadcast, therefore, multiple nodes may receive the BAM in the radius of provider node, thus result in broadcast storm. This flooding result in bandwidth wastage, battery issue of the wireless network, delay, redundancy, and so on.

Our second benchmark scheme is NDN-BBCD [40] which is a broadcast transmission mechanism for content delivery. In NDN-BBCD mobility-based forward node selection mechanism is proposed which tends to select less mobility nodes as forward ones. Moreover, an available link capacity-based forwarding scheme is used which tends to select nodes with larger link capacities to forward packets. The scheme has two main phases; content Discovery and content delivery. First nodes discover the provider nodes via maintaining a distance table (DT) which maintains the information of discovered providers. After that the node then select the best forwarder nodes. The purpose is to select those nodes which are less mobile and has high available link capacity. In this way, it not only reduces the control messages in the network but also reduce the number of nodes in the network that take part in the content delivery procedure. As the network is mobile and the provider may change its location frequently, therefore, it is highly likely that content discovery process may repeat many times. This content discovery process takes longer time and the DT is also an overhead in the wireless environment. For comparison purpose of NDN-BBCD, we have made changes in the strategy class of NFD. We have added a data structure which maintains the distance information of nodes in a table form. Therefore, whenever, a packet is received in the forwarder class of NFD, it calls the strategy class which maintains the DT. Furthermore, it is the responsibility of strategy class to select the nodes which have high link capacity and forward the packets after random defer time. The performance comparison of both benchmark protocols is discussed in the following subsections.

#### 6.3.1. Total Number of Data Packets Processed in Static Environment

**Figure 7:**
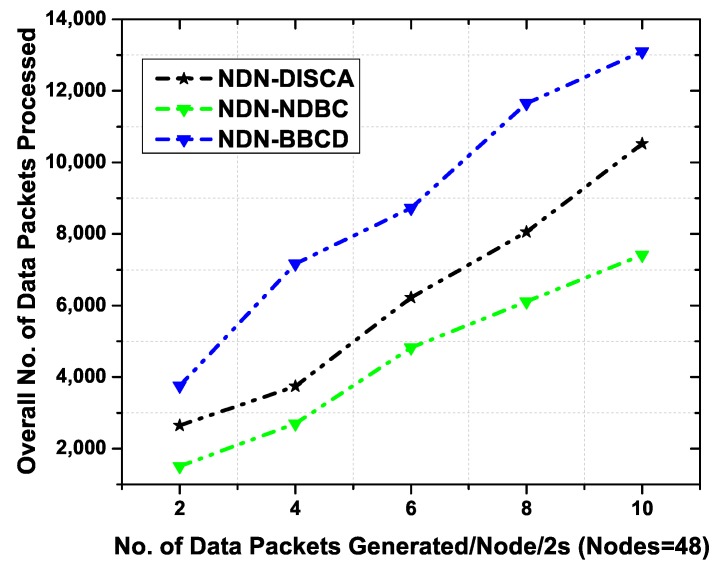
Overall number of data packets processed as a function of number of data packets generated per 2 s with 48 static nodes.

We evaluate the performance of the proposed NDN-NDBC protocol, NDN-DISCA, and NDN-BBCD by varying the number of data packets per 2 s in the network. Figure 7 shows the total number of data packets processed as a function of number of data packets generated per 2 s. The NDN-BBCD shows higher number of data packet transmission in the network than proposed NDN-NDBC protocol and NDN-DISCA. The reason is that the NDN-BBCD is based on broadcast transmission and pull-based mechanism. An interest message is broadcasted for data delivery in the network. This interest broadcast creates additional copies of the packets in the network which eventually results in the network congestion. Moreover, the NDN-DISCA, also shows higher number of data packets transmission than our proposed scheme i.e., NDN-NDBC. This is because the beacon alerts are broadcasted in the campus (up to 3 building in our case). Each node in each building broadcasts the beacon packets and then the data packets in the transmission range, thereby results in data packet broadcast storm. It is efficient than NDN-BBCD because of no interest packet transmissions. No special interest packets are transmitted for data delivery except the beacon messages. Therefore, the performance of NDN-DISCA is comparatively better than NDN-BBCD. In NDN-NDBC, the number of data packets processed in the network are smaller than the NDN-DISCA. The reason is the data broadcast Control mechanism which limits the data packets to a specific building defined by the content namespace and the device namespace in that building. All the devices discard the data packets if their namespace does not match the data namespace and hence the data packet flooding is controlled in the network. Simulation results show that our proposed scheme NDN-NDBC outperforms the NDN-DISCA and NDN-BBCD and enhance the performance of the network by controlling the extra data packets in the network.

#### 6.3.2. Total Number of Data Packets Processed in Mobile Environment

In this experiment, we have evaluated the performance of NDN-NDBC, NDN-BBCD, and NDN-DISCA by varying the number of data packet per 2 s in the network. We have used 48 static nodes and vary the number of mobile nodes from 2 to 4. As the static nodes only broadcasts the data packets in their transmission range, therefore, the data packets transmissions are low as compared to the mobile nodes. The mobile nodes move in the buildings for carrying data packets to appropriate consumers. Hence the data broadcasting increases with mobility of nodes in different regions and exhibit more data packets.

**Figure 8:**
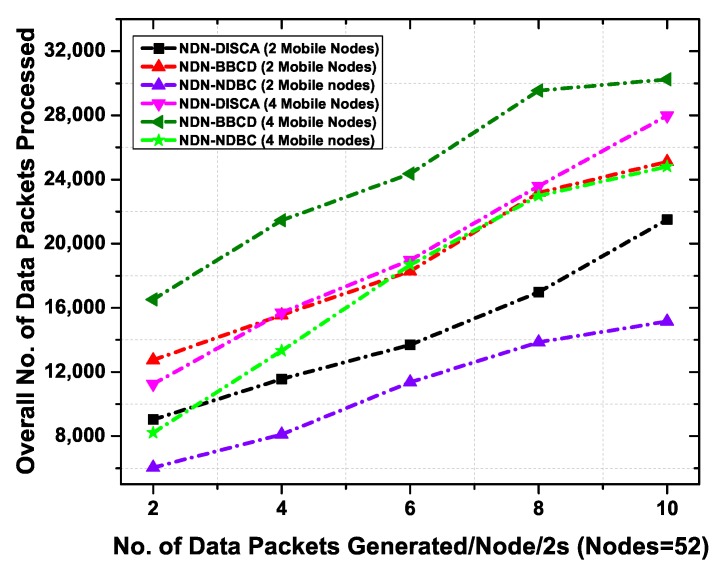
Overall number of data packets processed as a function of number of data packets generated per 2 s with 48 static nodes and (2–4) mobile nodes.

Figure 8 shows the total number of data packets processed as a function of number of data packets generated per 2 s in the network. The NDN-DISCA and NDN-BBCD shows higher number of data packets than our proposed scheme i.e., NDN-NDBC. This is because the NDN-DISCA first generates beacon alerts and then broadcast in the network. Moreover, NDN-BBCD uses interest and data exchange for content delivery which results in extra copies of packets in the network. Whereas in NDN-NDBC, the data packets are controlled to a specific building where the consumer resides. In NDN-DISCA and NDN-BBCD the mobile nodes (2 and 4) are moving in the building for carrying data packets. These mobile nodes when come in the range of other buildings, then the devices of other buildings also broadcast the data packets. As a result, more data packets are generated in the network and creates data broadcast storm. The NDN-DISCA outperforms the NDN-BBCD due to the reason of no interest packet transmission and rely only on the beacon message for data delivery. However, NDN-BBCD rely on the interest and data exchange process for content discovery and delivery. In NDN-NDBC, all the nodes including mobile nodes after receiving the data packet first check the namespace of the data packet. All the devices discard the data packets if their namespace does not match the data namespace and hence the data packets flooding are controlled in the network. From this experiment, we have concluded that the mobile nodes result in more data packets transmission as compared to static nodes. In the case of NDN-DISCA and NDN-BBCD, the data broadcast becomes higher with increasing number of mobile nodes in the network. However, in NDN-NDBC the number of data packets transmission is mitigated in the network. This proves that if the data is controlled in a specific region then it results in alleviating traffic on the network, and less network resources are used.

#### 6.3.3. Total Energy Consumption in Static Environment

In this experiment, we investigate the performance of the proposed NDN-NDBC protocol, NDN-BBCD and NDN-DISCA by varying the number of data packets per 2 s in the network and have used 48 static nodes. Due to high data packets transmissions in the network, the overall energy consumption of nodes and bandwidth use is affected, overall. The reason is that the size of data packets is larger than the interest packets and remarkably impact the network resources. In NDN-DISCA, all the intermediate nodes first generate an alert message and then broadcast it in the network. As the beacon messages carry control information, therefore, broadcasting of beacon messages may increase the chances of network congestion and more resources may be used. Moreover, in NDN-BBCD more packets are transmitted than NDN-DISCA due to interest and data exchange for content discovery and content delivery. As a result, a higher amount of energy is consumed in the network. Whereas the proposed scheme NDN-NDBC does not rely on beacon message nor interest messages and forwards a smaller number of data messages, overall. The reason behind this phenomenon is many nodes simply discard the data messages that have not a match for data packet namespace with their node id.

**Figure 9:**
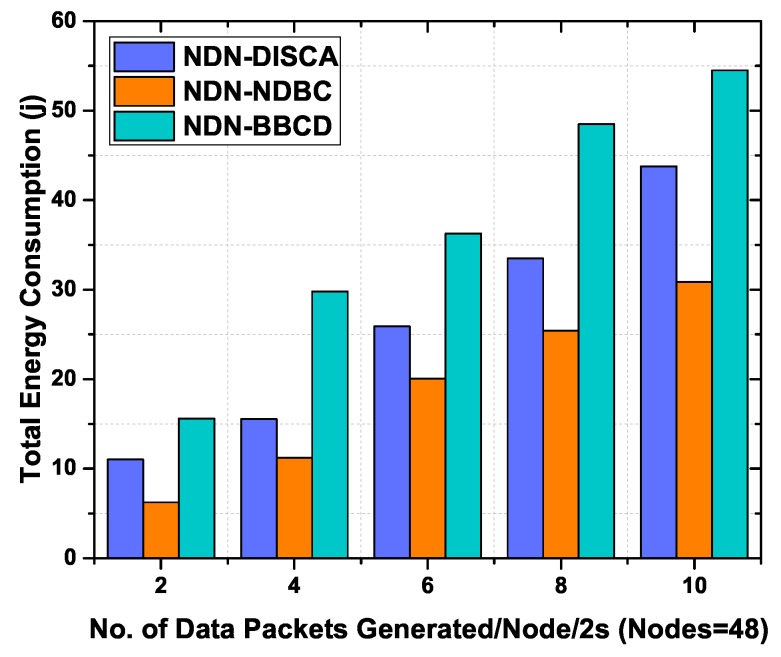
Total energy consumption as a function of number of data packets generated per 2 s with 48 static nodes.

Figure 9 depicts the total energy consumed by all static nodes in relationship with the data generate rate. The proposed scheme NDN-NDBC outperforms NDN-BBCD and NDN-DISCA in terms of total energy consumption for various number of data packet generation rate. It is obvious that the energy consumption has a strong relation with the number of data messages processed in the network and the large size of data packets results in more energy consumption. It is also evident from the previous discussion that the proposed scheme minimizes the number of data packets in the network. Hence it saves the battery resources overall in the network.

#### 6.3.4. Total Energy Consumption in Mobile Environment

**Figure 10:**
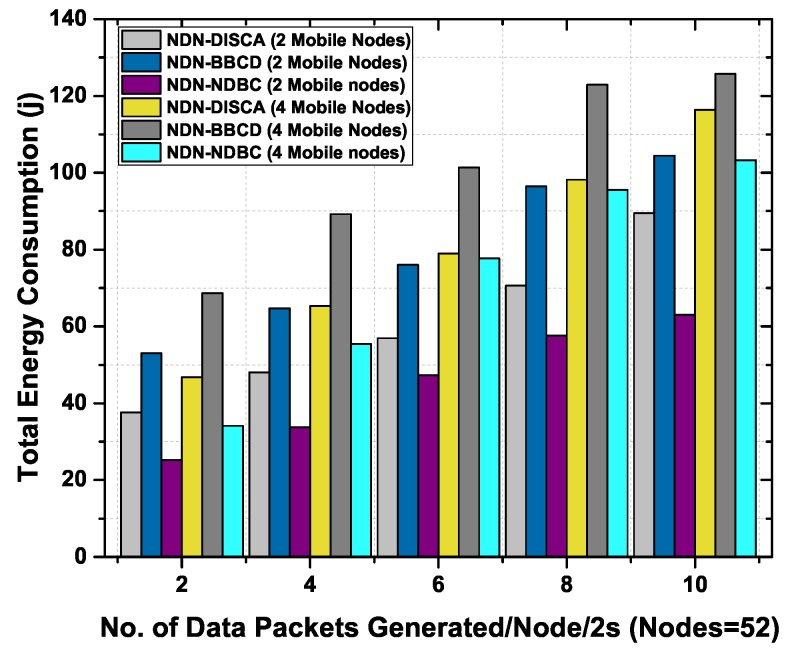
Total energy consumption as a function of number of data packets generated per 2 s with 48 static nodes and (2–4) mobile nodes.

We also analyze the energy consumption of the proposed NDN-NDBC scheme with NDN-BBCD and NDN-DISCA by varying the number of mobile nodes in the network from 2 to 4. Figure 10 shows the total energy consumption of all nodes in the network as a function of number of data packets generated per 2 second with 2 and 4 mobiles nodes. The mobile nodes exhibit more data packets while moving in the network. It is evident that NDN-DISCA and NDN-BBCD shows high power consumption in both case of mobile nodes (2 and 4). The reason is the number of control information are used for data delivery in NDN-BBCD and beacon messages in NDN-DISCA. Whereas NDN-NDBC shows less energy consumption than NDN-DISCA and NDN-BBCD in both case of mobile nodes (2 and 4). The is due to the reason the data packets are controlled to a specific building where the consumer resides. The proposed scheme NDN-NDBC controls the broadcast storm in the network including mobile nodes and discards the data packets which do not belong to a data namespace. As we have discussed before the data packet directly impacts the energy consumption of the nodes. Therefore, the total energy consumption of NDN-NDBC protocol is less than NDN-DISCA and NDN-BBCD due to controlled data packets.

#### 6.3.5. Average Delay

**Figure 11:**
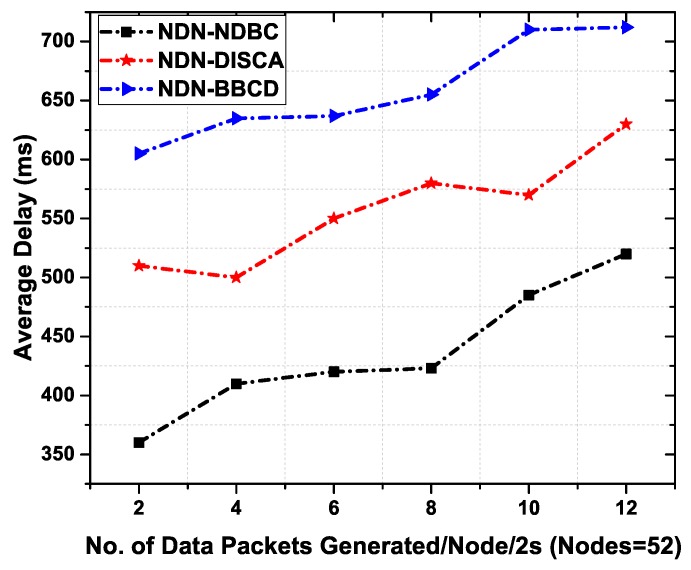
Average Delay as a function of number of data packets generated per 2 s.

Figure 11 depicts the average delay of NDN-BBCD, NDN-DISCA, and NDN-NDBC scheme in a building scenario. NDN-BBCD shows a higher delay than the other two schemes and we have noticed that overall transfer time of data packet in NDN-BBCD is twice or thrice that of proposed scheme. This is due to the reason that in the NDN-BBCD, the mechanism is request response using DT for delivering data packets. The delay for NDN-BBCD includes the time a request packet (REQ) is generated, the delay for response packet (RSP), the delay for the data packet request (REQD) and the delay for data delivery. The NDN-DISCA consumes slightly less delay than NDN-BBCD. The reason is in NDN-DISCA, the delay includes the time for beacon alert message and data delivery delay only. The proposed NDN-NDBC outperform other schemes due to the reason that we are not using any beacon messages, REQ, and RSP. The data is controlled in a specific region of a building where it is destined.

### 6.4. Vehicular NDN Scenario and Experimental Setup

To evaluate our scheme NDN-NDBC, we consider a VNDNs scenario through an ndnSIM simulator. We compare our scheme with the most recent protocols i.e., NDN-PCDF “Enabling Push-Based Critical Data Forwarding in Vehicular Named Data Networks” [37] and NDN-CODIE “CODIE: Controlled Data and Interest Evaluation in Vehicular Named Data Networks” [6]. We implemented NDN-CODIE and NDN-PCDF for our evaluations. The changes are made in the strategy class of NFD. The total simulation time is 1200 s and the data packet size is 1040 bytes. In simulation, we consider three RSUs and 50 number of intermediate nodes (vehicles) and one producer node (which generates critical data packets). The average speed of vehicles is 60 kmph. To exhibit a realistic scenario and for spreading all vehicles in the network, the data packet transmission started after 3 s of the simulation began.

#### 6.4.1. Performance Analysis

In NDN-PCDF critical data transfer is enabled via a beacon messages support for VNDNs. The beacon messages are used to create synthetic entries in PIT table for critical data whose entries are not created before. However, the beacon messages are forwarded in a broadcast manner towards the destined consumer. Due to broadcast nature of wireless medium the beacons messages are broadcasted and may be received by other RSUs in the range. Although the beacon messages are of small sizes, it also causes congestion and waste the bandwidth and other resources of the network.

In NDN-CODIE the number of data copies are reduced in a network by using a hop count to limit the data packet propagation. The NDN-CODIE also uses the interest and data exchange mechanism and each node in NDN-CODIE while forwarding an interest packet including hop counter h. If the intermediate node is not a provider node then it increments the h, create PIT entry and forward the interest packet. When the provider node receives the interest packet, the provider increments h and includes the latest value in the data dissemination limit (DDL) field in data packet. This DDL field limit the copies of the data packets in the network. Since this scheme rely on interest transmission, hence it creates more packet in the network than NDN-PCDF and our proposed scheme NDN-NDBC which do not rely on the transmission of interest packets.

In the following text, we represent the performance analysis of our scheme NDN-NDBC with NDN-PCDF and NDN-CODIE.

#### 6.4.2. Total Number of Data Packets Processed with Data Generation Rate

**Figure 12:**
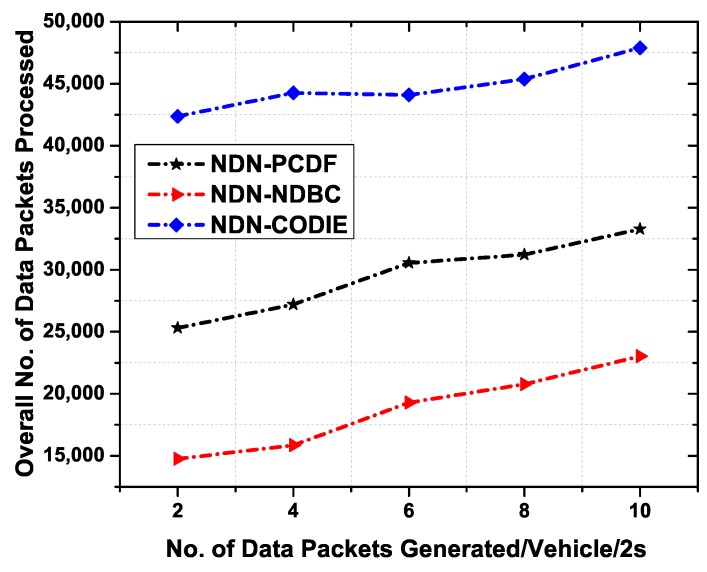
Overall number of data packets processed as a function of number of data packets generated per 2 s.

In this phase, we evaluate and conduct experiment to check the performance of the proposed scheme NDN-NDBC as well as the benchmark scheme NDN-CODIE and NDN-PCDF against the number of data packets generated per vehicle per 2 s. Total number of nodes are 50 and the average speed is 60 Kmph. It is evident from Figure 12 that NDN-NDBC remarkably decreases the number of data packets in the network comparatively than NDN-CODIE and NDN-PCDF. This is achieved due to name-based data control. In the NDN-PCDF case, all the vehicles no matter which RSU they belong to, first broadcast a beacon message and then forwards the data packets which result in broadcast storm in the network. Moreover, in NDN-CODIE, first an interest message is generated for content and then the data packet comes back up to h number of hops recorded in the DDL field. This mechanism includes extra data structure which eventually increases the overhead in the network. Hence the overall number of data packets processed in NDN-CODIE are higher than the other two schemes. However, in NDN-NDBC, the vehicles in the range of RSU when receive the data packet, first compare their namespace with the received data packet namespaces. If the data packet is destined for that vehicle, then it processes the data packet, otherwise, all the data packets are discarded and are not processed/forwarded further.

#### 6.4.3. Total Number of Data Packets Processed with Relative Speed

**Figure 13:**
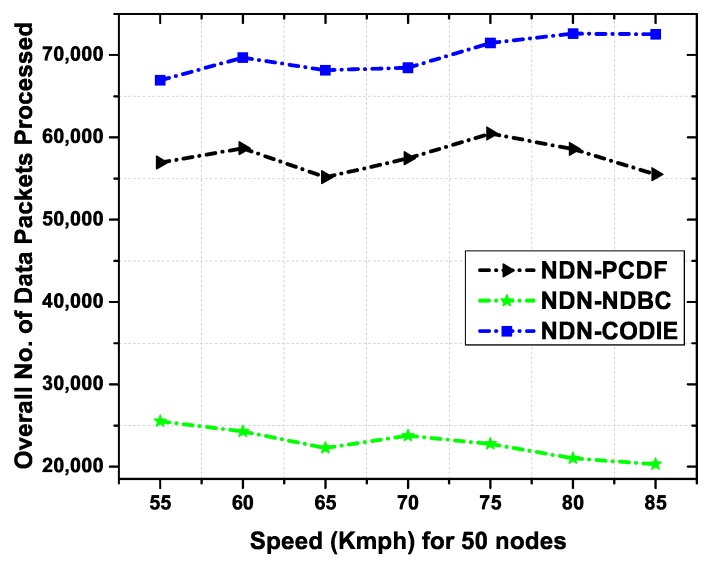
Overall number of data packets processed as a function of vehicle speed.

To analyze the behavior of VNDNs, we explore the performance of the proposed NDN-NDBC scheme NDN-CODIE and NDN-PCDF scheme by considering the more challenging environment (e.g., vehicle speed). Figure 13 depicts the total number of data packets processed as a function of the vehicle speed. The proposed NDN-NDBC scheme significantly decreases the total number of data packets in the network. This is because the intermediate vehicles process the data packets based on corresponding names of vehicles in a certain region of RSU provided by GPS. If a relay vehicle is not in the region of an RSU for which the data packet is destined, it simply discards the data packet. This phenomenon reduces the unnecessary flooding in the network. Moreover, the dynamic environment of VNDNs does not allow long-lasting connections and the vehicle may move very quickly to other region thus results in low data packets processing. As we increase the vehicle speed, the overall trend is decreasing. This is due to the reason that vehicle stay in a specific region for a short time. As a result, a smaller number of packets will be processed in that region.

#### 6.4.4. Average Delay

**Figure 14:**
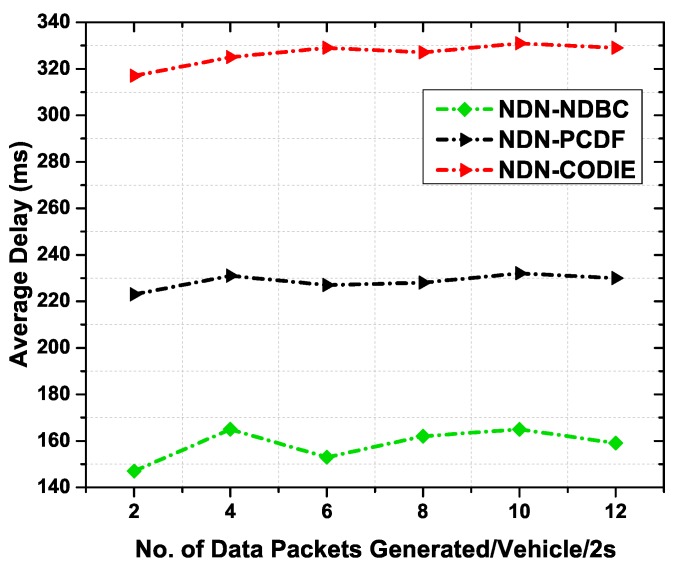
Average Delay as a function of number of data packets generated per 2 s.

Figure 14 illustrates the average delay of NDN-CODIE, NDN-PCDF, and NDN-NDBC scheme in a vehicular scenario. NDN-CODIE exhibits higher delay than the other two schemes. It is observed that NDN-CODIE uses higher bandwidth and thus increases congestion in the network. This is due to the reason that NDN- CODIE follows the interest and data exchange mechanism and the delay includes the time an interest is generated and the delay for the data packet delivery. This delay is defined as the round-trip time between interest and data retrieval. Figure 14 shows that NDN-CODIE faces higher delay than NDN-PCDF and NDN-CODIE. This is due to higher number of packets in the network that may cause an increase in congestion and collisions. Whereas NDN-PCDF shows less delay than NDN-CODIE due to beacon messages. Beacon messages are used for dissemination data in the network and no interest packets are needed in this case, thus results in a smaller number of packets in the network which eventually reduces delay. The proposed NDN-NDBC outperform other schemes due to the reason that NDN- NDBC is based on content names and device names for a destined user in a specific region. No interest messages and beacon messages are needed to deliver the content towards consumer. This reduces the number of packets in the network and increases the possibility of data packet to reach the consumer nodes with minimum delay.

## 7. Conclusions

NDN supports pull-based traffic; however, in case of an emergency, the critical data must be forwarded immediately without any consumer request for the data. Although solutions are provided to enable push-based traffic in NDN-based IoT, the existing literature lacks an efficient control of push-based data broadcast. Due to wireless broadcast nature, if the data packets are not controlled via an efficient mechanism then it may lead to high consumption of network bandwidth and may use a lot of resources of the network. Specifically, in case of push-based scenarios, the critical data may not reach to the destined consumers on proper time. To address this issue, we proposed a naming scheme that controls data packets in a specific region where the data packets are destined instead of sending to all the devices in a network. In this way, not only the resources of the network are saved, but the critical data may be reached to appropriate consumer at appropriate time. The proposed method is a hierarchical name-based scheme which comprises of a robust content namespace design, device namespace design, minor amendments to the data packet format and unsolicited data policy of forwarding engine as well. The proposed method controls the number of unnecessary data packets in the network via robust content namespace and device namespace design. We have employed our scheme in two most important use cases i.e., smart building and VNDNs. The simulation results show that the proposed scheme outperforms the benchmark schemes in terms of total number of data packets processed in the network, total energy consumption of nodes in the networks and average delay against the data generation rate and relative speed of vehicles. As a future work, we plan to apply our proposed scheme in VNDNs enabled edge cloud computing. In addition, we also plan to provide name-based interest control for VNDNs with edge cloud computing.

## Figures and Tables

**Table 1:** Simulation Parameters for Building Scenario.

Parameter	Value
Simulator	NS-3 (ndnSIM)
Propagation Delay Model	Constant-Speed Propagation
Propagation Loss Model	Log Distance
Mobility Model for static nodes	Random_Disc_Position_Allocator Model
Mobility Model for mobile nodes	Random_Direction 2D Mobility Model
Mobile Node speed	0.3 s
Technology	Wi-Fi_STANDARD_IEEE 802.11a
Area (m × m)	40 × 40
Number of Nodes	52 (48 Static Nodes and 4 Mobile Nodes)
Frequency of Data packets/2s	2, 4, 6, 8, 10
Packet Size	1040 bytes
TxPowerStart	5 (dbm)
TxPowerEnd	5 (dbm)
Energy Consumption	0.5 μJ/bit
Initial Energy	10 Joules
Caching Policy	LCE
Replacement Policy	LRU
Simulation Time (s)	1200 s
